# Child and adolescent mental health services: longitudinal data sheds light on current policy for psychological interventions in the community

**DOI:** 10.1108/JPMH-03-2017-0013

**Published:** 2017-12-18

**Authors:** Sharon A.S. Neufeld, Peter B. Jones, Ian M. Goodyer

**Affiliations:** Department of Psychiatry, University of Cambridge, Cambridge, UK

**Keywords:** Policy, Depression, Child and adolescent mental health services, Community interventions

## Abstract

**Purpose:**

The purpose of this paper is to expand upon policy implications of a recent study assessing adolescent mental health service contact and subsequent depression.

**Design/methodology/approach:**

Review of related evidence from academic and grey literature.

**Findings:**

Studies assessing the role of mental health services in reducing mental disorder during adolescence are sparse, and even prevalence figures for adolescent mental disorders are out-of-date. Adolescent mental health service contact rates are shown to fall concurrent with budgetary decreases. School-based counselling is highlighted as an important source of help that may be at risk of being cut. Increased training of General Practitioners and school counsellors is needed to improve efficiency in specialist Child and Adolescent Mental Health Services (CAMHS).

**Practical implications:**

Longitudinal studies of young people’s mental health should include mental health service usage and its relationship with subsequent mental health outcomes.

**Social implications:**

Funding cuts to CAMHS must be avoided, school-based counselling must be protected, and service referrers should be better trained.

**Originality/value:**

This paper highlights the need for increased CAMHS data, sustained funding, and improved training for this vital service.

Young people’s mental health problems account for many adulthood adversities, including greater likelihood of mental disorder (Jones, 2013), decreased income, decreased probability of being employed or maintaining a stable cohabiting relationship (Goodman *et al.*, 2011), and greater contact with the criminal justice system (Knapp *et al.*, 2016). Increasing the effectiveness and numbers treated by Child and Adolescent Mental Health Services (CAMHS) would therefore yield personal, economic, and societal benefits over the lifespan. Recently, a paper was published supporting the association of treatment-as-usual mental health service contact with improved mental health by late adolescence (Neufeld *et al.*, 2017). While the baseline mental health service data were collected a decade ago (2005/2006), such data are rare and provide insights relevant to current CAMHS. Some policy implications for CAMHS arise from this study, pertaining to the evidence base, funding, continuity of services, and training of referrers.

Studies assessing the role of mental health services in reducing mental disorder during adolescence are sparse, an oversight that must be addressed. The literature review conducted by Neufeld *et al.* (2017) found only six studies internationally which assessed the relationship of adolescent mental health services and subsequent mental health; none were as rigorous in simultaneously addressing non-randomisation of service usage, attrition, and clinical relevance as Neufeld *et al.* (2017). Longitudinal studies of young people’s mental health should without question include mental health service usage and its relationship with subsequent mental health outcomes. Policy would be more greatly informed if larger samples (e.g. national surveys) assessed a variety of psychosocial and perhaps physiological outcomes by individual diagnoses and/or treatment sectors. In the UK, such data are sorely lacking. Even up-to-date prevalence figures for mental disorders are glaringly absent: the most recent survey was last carried out in 2004 (Green *et al.*, 2005). Whilst a new survey will be carried out in 2017 (HM Government, 2017), the lag in collecting information that so vitally instructs service provision and planning for young people is concerning. For comparison, the Adult Psychiatric Morbidity Survey has been conducted twice as often – every seven years (McManus *et al.*, 2016). National surveys on young people’s mental health must keep pace with those performed in adults, and they must rigorously assess the impact of service contact.

The data from the Neufeld *et al.* (2017) study were obtained prior to funding cuts to CAMHS, and taken together with other evidence, can make the case for how deleterious such austerity is to mental health service access for young people. From 2005/2006, Neufeld *et al.* (2017) found that 38 per cent of 14-year olds with a mental disorder had made contact with mental health services in the past year; however, in 2014/2015 only 25 per cent of all children and young people with a mental disorder had made such service contact (NHS England, 2015). During this time, between 2008/2009 and 2012/2013, CAMHS funding dropped by 5.4 per cent in real terms (Lamb, 2015) so that in 2012/2013, only 6 per cent of NHS’ total mental health budget was spent on CAMHS (McShane *et al.*, 2015). Services from data in 2005/2006 that Neufeld *et al.* (2017) showed were related to an improvement in subsequent depression in young people have been overstretched due to austerity. For example, the number of young people attending A&E due to a psychiatric condition had more than doubled in 2014/2015 compared with 2010/2011 (Frith, 2017), indicating a breakdown in access to primary mental health services. In contrast, funding for adult mental health services was less impacted during this period, with NHS funds falling for the first time in a decade in 2011/2012 by 1 per cent in real terms (The Kings Fund, 2015). Encouragingly, among adults with mental disorder, service contact rose from 24 per cent in 2007 to 37 per cent in 2014 (McManus *et al.*, 2016), indicating that more stable funding can facilitate service access. It is heartening that the NHS aims to increase rates of young people’s mental health service contact back up to 35 per cent by 2020/2021 (NHS England, 2015). However, society must acknowledge the suffering in our young people that has not been alleviated due to austerity measures, and resolve to ever-increase connection of young people with mental health services which are effective.

Data from the Neufeld *et al.* (2017) paper indicate the importance of school-based counselling, yet this source of help for young people must be protected. Neufeld *et al.* found that for those with a mental disorder, after specialist CAMHS, the next most used service was school counselling, and for those without a mental disorder, school counselling was the most highly used service. The current government has promised to provide funding for mental health first aid training for teachers in secondary schools (HM Government, 2017), enabling them to better identify those with mental health issues and connect them to support services (Mental Health First Aid England, 2016). However, this is against a backdrop of freezing school budgets until 2020/2021, the very budgets which typically fund school-based counselling (Frith, 2016). Increased identification of young people’s mental health problems is commendable; at the same time leaving key services at risk of being cut is highly counterproductive, potentially increasing pressure on more specialist CAMHS. In total, 90 per cent of the cost of young people’s mental health problems falls on the education system (Frith, 2017). The fact that young people who do not meet diagnostic criteria are referred back from specialist CAMHS to counselling in schools and General Practitioner (GPs) surgeries (Frith, 2016) underscores the importance of such services in preventing more serious problems. Funding for school-based counselling must be ring-fenced, whether it be funded through the education sector or NHS, to ensure young people have adequate service access prior to specialist CAMHS.

Service referrers, such as those in primary care or schools, need to be better trained in identifying the presenting features of mental disorders, to help prioritise specialist CAMHS for more serious cases. Neufeld *et al.* (2017) showed that improvements related to mental health service contact were only seen in those who had a clear need for services, as defined by the presence of a mental disorder. The findings imply that those meeting a diagnostic threshold of mental disorder may be more responsive to treatment, and that prioritising more serious cases could make the system more effective. Currently, specialist CAMHS turn away 23 per cent of the children and young people referred to them for treatment by GPs or teachers (Frith, 2017). This implies two things: specialist CAMHS cannot cope with the population needs, and/or referrals need to be more appropriately made. Both may be true. Regardless, increased training of GPs and school counsellors could improve efficiency in specialist CAMHS by minimising subthreshold cases that are assessed but not uptaken by CAMHS. Such efficiency is sorely needed particularly when services are overburdened, and could help improve waiting times, which have been found to be unacceptably long (Frith, 2017). Indeed, it is clear that GPs could use more training in identifying mental disorders. The Royal College of General Practitioners (2016) reports that 90 per cent of people with mental health problems are managed in primary care. However, even in the recent past, most GP training has not included a rotation in mental illness (The Centre for Economic Performance’s Mental Health Policy Group, 2012). Such gaps in training do nothing to mitigate the treatment gap: a meta-analysis showed that GPs correctly identified only 47.3 per cent of depression cases (Mitchell *et al.*, 2009). A high rate of access to individuals with mental disorders coupled with insufficient background knowledge to appropriately identify such cases represents an egregious missed opportunity. In addition to improved training for GPs, there should also be a clear pathway for training and supervision of school-based psychological workers to facilitate appropriate referrals to specialist CAMHS from the education sector. Care needs to be taken to ensure strong connections with primary care and schools to specialist CAMHS for young people who need additional help.

In sum, while the association of mental health services with a subsequent decline in adolescent depression (Neufeld *et al.*, 2017) is heartening, more studies of this nature are needed. Larger samples could enable a better understanding of the relative roles of various sectors in reducing specific mental disorders, to more specifically inform service provision. Neufeld *et al.*’s (2017) data suggest funding cuts have drastically reduced rates of service contact, and that school-based counselling is a well-utilised service, which may be at risk of being cut when the data suggest it should not be. Ensuring this service and better training of service referrers could help ease the strain on specialist CAMHS, and help all CAMHS work in a more integrated fashion.
